# The complete chloroplast genome of *Cnidium officinale* Makino

**DOI:** 10.1080/23802359.2018.1464413

**Published:** 2018-04-23

**Authors:** Inkyu Park, Sungyu Yang, Wook Jin Kim, Pureum Noh, Hyun Oh Lee, Byeong Cheol Moon

**Affiliations:** a K-herb Research Center, Korea Institute of Oriental Medicine, Daejeon, Republic of Korea;; b Phyzen Genomics Institute, Seongnam, Republic of Korea

**Keywords:** *Cnidium officinale*, *Ligusticum officinale*, medicinal plant, chloroplast genome, Apiaceae

## Abstract

*Cnidium officinale (Ligusticum officinale)* is an important herbal medicine. To facilitate species identification, we determined the complete chloroplast genome of *C. officinale* using the Illumina MiSeq platform. The genome was 148,518 bp in length, comprising a large single copy (LSC) region of 93,977 bp, a small single copy (SSC) region of 17,607 bp, and two inverted repeat regions (IRa and IRb) of 18,467 bp each. The genome contains 113 unique genes, including 79 protein-coding genes, four ribosomal RNAs (rRNAs), and 30 transfer RNAs (tRNAs). Phylogenetic analysis revealed that *C. officinale* is most closely related to *L. tenuissium*, with high bootstrap values.

*Cnidium officinale*, a member of the Apiaceae family, is widely distributed in China and Korea. The dried rhizomes of *C. officinale* are used as a herbal medicine, called Cnidii Rhizoma, in East Asia (KIOM 2016); the medicine is used to relieve pain and treat vitamin deficiency disease, menstrual disturbance, inflammation, and hypertension (Lee et al. 2016). Although the dried rhizomes of *C. officinale* are medicinally valuable, Cnidii Rhizoma is frequently mixed and mis-used indiscriminately with other similar species when sold in Korean herbal markets. To facilitate identification and discrimination from other species, we sequenced the chloroplast genome of *C. officinale*.

We collected fresh leaves of *C. officinale* from medicinal plantations in Korea (36°48′01.9″N 128°57′47.2″E). Specimens were given identification numbers and registered in the Korean Herbarium of Standard Herbal Resources (Index herbariorum code KIOM) at the Korea Institute of Oriental Medicine (KIOM), with Voucher no. KIOM201501014665. Genomic DNA was extracted using the DNeasy Plant Maxi kit (Qiagen, Valencia, CA). An Illumina paired-end library was constructed and sequenced using the MiSeq platform (Illumina Inc., San Diego, CA). Sequencing yielded ∼2 Gb of high-quality paired-end reads.

The chloroplast contigs of *C. officinale* were *de novo* assembled from low-coverage whole-genome sequences. The complete chloroplast genome is 148,518 bp in length (GenBank Accession no. MH121055) and has a typical quadripartite structure, consisting of a large single copy (LSC) region of 93,977 bp, a small single copy (SSC) region of 17,607 bp, and two inverted repeat regions (IRa and IRb) of 18,467 bp each. The GC content is 37.6%, with the IR regions having higher GC content (44.8%) than the LSC (36%) and SSC (31.1%) regions. Thus, the *C. officinale* chloroplast genome is AT-rich, similar to other chloroplast genomes (Qian et al. 2013; Park et al. 2017). The genome contains 113 unique genes, including 79 protein-coding genes, 30 transfer RNAs (tRNAs), and four ribosomal RNAs (rRNAs). Seventeen of the genes are duplicated in IR regions.

To investigate the phylogenetic relationships of *C. officinale*, we aligned 59 protein-coding gene sequences with their homologs from 13 other taxa. The maximum likelihood (ML) tree of nine nodes had bootstrap values of 100% (Figure 1), i.e. the phylogenetic relationship was well supported within the family Apiaceae. The ML tree revealed that *C. officinale* forms a monophyletic group with *L. tenuissium* in tribe Selineae, with bootstrap support values of 100% (Figure 1).

**Figure 1.**
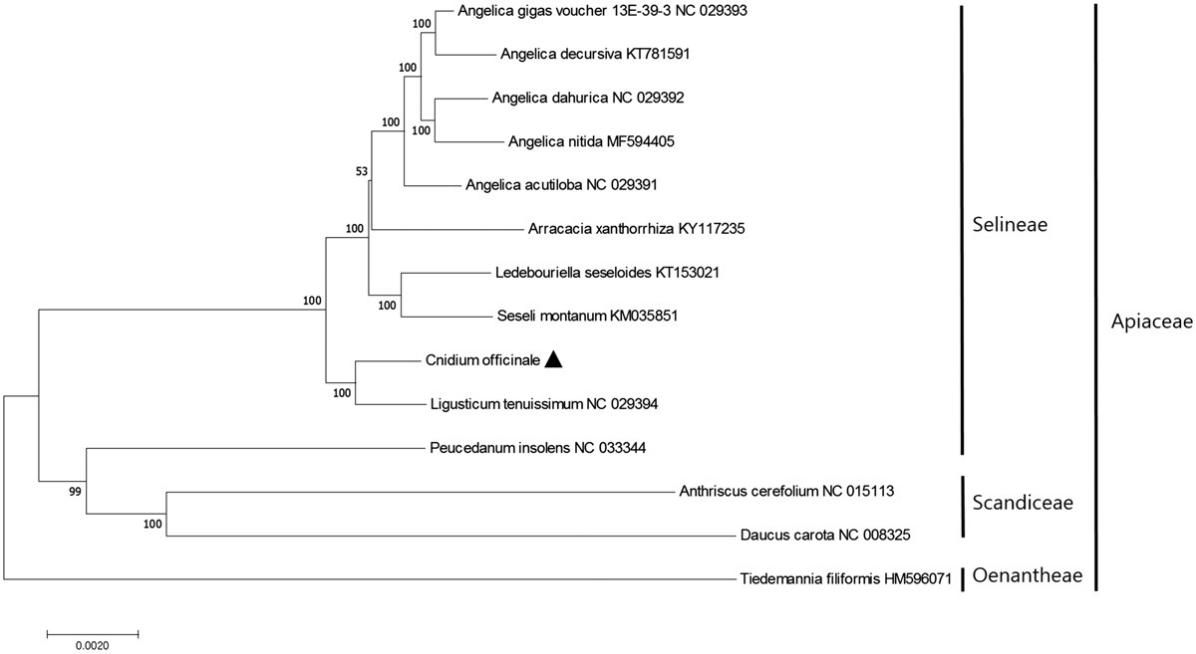
Maximum-likelihood (ML) tree based on the chloroplast protein-coding genes of 14 taxa, including *C. officinale* and one outgroup taxon. Fifty-nine protein-coding genes were aligned using MAFFT (http://mafft.cbrc.jp/alignment/server/index.html). ML analysis was performed using MEGA6, with 1000 bootstrap replicates (Tamura et al. 2013). The bootstrap support values from 1000 replicates are indicated at the nodes.
